# Mental disorders in Bangladesh: a systematic review

**DOI:** 10.1186/s12888-014-0216-9

**Published:** 2014-07-30

**Authors:** Mohammad Didar Hossain, Helal Uddin Ahmed, Waziul Alam Chowdhury, Louis Wilhelmus Niessen, Dewan Shamsul Alam

**Affiliations:** Centre for Control of Chronic Diseases (CCCD), International Centre for Diarrheal Disease Research, Bangladesh (icddr,b), 68 Shaheed Tajuddin Ahmed Sharani, Mohakhali, Dhaka, 1212 Bangladesh; National Institute of Mental Health (NIMH), Sher-E-Bangla Nagar, Dhaka, 1207 Bangladesh; Liverpool School of Tropical Medicine, Pembroke Pl, Liverpool, Merseyside L3 5QA UK; Johns Hopkins Bloomberg School of Public Health, Baltimore, MD USA

**Keywords:** Mental disorders, Depression, Systematic review, Prevalence, Comorbidity, Service delivery, Management, Treatment, Bangladesh

## Abstract

**Background:**

Mental disorders constitute a major public health problem globally with higher burden in low and middle-income countries. In Bangladesh, systematically-collected data on mental disorders are scarce and this leaves the extent of the problem not so well defined. We reviewed the literature on mental health disorders in Bangladesh to summarize the available data and identify evidence gaps.

**Methods:**

We identified relevant literature on mental disorders within Bangladesh published between 1975 and October, 2013 through a systematic and comprehensive search. Relevant information from the selected articles was extracted and presented in tables.

**Results:**

We identified 32 articles which met our pre-defined eligibility criteria. The reported prevalence of mental disorders varied from 6.5 to 31.0% among adults and from 13.4 to 22.9% among children. Some awareness regarding mental health disorders exists at community level. There is a negative attitude towards treatment of those affected and treatment is not a priority in health care delivery. Mental health services are concentrated around tertiary care hospitals in big cities and absent in primary care.

**Conclusions:**

The burden of mental disorders is high in Bangladesh, yet a largely unrecognized and under-researched area. To improve the mental health services in Bangladesh, further well-designed epidemiological and clinical research are needed.

## Background

Mental disorders constitute a major public health problem and contribute to 13% of the global burden of disease measured as disability adjusted life years [1]. Low and middle income countries have higher burden of mental disorders than economically developed countries [2,3]. Mental disorders have serious negative effect on survival, and when present with chronic diseases as co-morbid condition, serious mental disorders may reduce life expectancy by about 20 years [4]. Mental disorders are generally not perceived as a health problem and are not priority in the health care delivery. Epidemiological and health system data related to mental disorders are scarce and are not readily available in Bangladesh although a few published articles provide some estimates of different mental disorders.

This review was conducted to understand the prevailing situation and trends in mental disorders in Bangladesh. This is expected to generate useful insights and may assist health professionals and policy makers in defining the need and planning service delivery models.

## Methods

### Search strategy

We searched, collected and evaluated literature on mental disorders based on the Preferred Reporting Items for Systematic Reviews and Meta-Analyses (PRISMA) checklist [5]. The PRISMA protocol was chosen from several methodologies and guidelines for the optimal reporting of systematic reviews specifically for quantitative studies. We followed the PRISMA checklist [5] for the extraction and tabulation of information. We identified relevant community and facility based literature through a comprehensive scientific literature search using the data-bases of PubMed and the Bangladesh Journals Online. We used the following search terms: “mental disorders, depression, prevalence, comorbidity, service delivery, referral, management, treatment, Bangladesh”. We combined search terms using Boolean operators to narrow the search results. We carried out a manual search to identify additional articles was carried out based on the bibliographies of the identified published studies (‘snowballing’). References and cross-references of the articles were critically studied through manually to find any relevant study missed by the electronic and/or manual search. Additionally, local journals that could not be accessed online were also searched manually. We also retrieved the full-text for the unpublished/gray literature from the library and Information Services Unit (LISU) of the icddr,b and library of the National Institute of Mental Health (NIMH) Bangladesh. Citations were managed using EndNote version X7.0.2. A narrative synthesis of the finally selected articles was reported.

### Inclusion and exclusion criteria

We included articles which presented (i) quantitative outcome data on mental disorders among Bangladeshi population, (ii) reported on human participants and (iii) were published between 1975 (earliest listed publication in Bangladesh) and October, 2013 (latest publication obtained) and (iv) published in English. We excluded articles which were: (i) qualitative studies, (ii) those published as theses/dissertations, and (iii) not in English.

### Quality assessment

Initially, two authors (MDH & DSA) screened and evaluated each article individually to decide on its inclusion or exclusion. Articles were further assessed for (i) the appropiateness and clarity of the research question/objectives/aims (y/n ) and the study design chosen (y/n), (ii) adequate description of study location (y/n), sample/participants (y/n), data collection methods (y/n), context of collection and quantitative outcome data presented (y/n) (iii) adequacy of measurement and appropiateness of statistical analysis (e.g. the odds ratio, *p* values and confidence interval) (y/n). For each article found, titles and abstracts were initially examined to determine whether the selection criteria were met. If an article failed to meet these criteria, the full text article was not retrieved and was excluded. In case of any disagreement on quality assessment checklist, three authors (MDH, HUA & DSA) discussed together and reached an agreement about inclusion or exclusion of that particular article.

**Figure 1:**
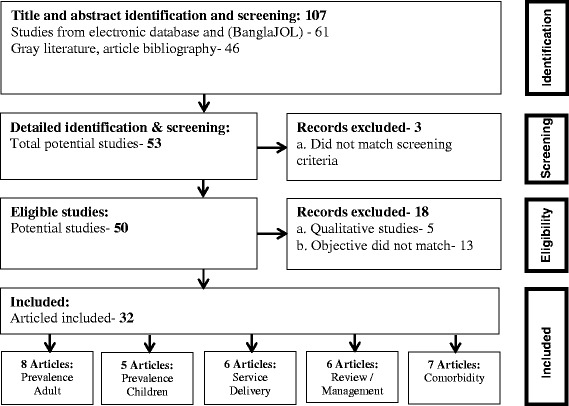
**An adapted PRISMA flow diagram of the literature selection process for inclusion in the systematic review.**

We categorized the articles and tabulated by (1) study location: “rural and urban” (2) study method: “prevalence/cross-sectional study, case control, cohort, specific population survey and trials”, and by (3) outcome: “mental disorders, adult prevalence, child prevalence, service delivery/referral pattern/management/treatment, types of co-morbidity”. A record of all excluded studies and the reasons for exclusion was documented. The selection process of the articles is displayed in Figure 1.

## Results

### Selection of literature

Through the initial search of databases, we identified 107 articles on mental disorders in Bangladesh. After the review of titles and the abstracts we excluded 54 articles as they were deemed not relevant to the review. Out of the remaining 53 articles, 3 failed to meet the screening criteria and full text of the remaining 50 articles were further reviewed and checked for eligibility which resulted in further exclusion of another 18 articles, 5 of which were due to qualitative in nature [6–10] and 13 did not fulfill the required methodological criteria. Finally, 32 studies met the inclusion criteria for the review (Figure 1). Most common mental disorders in the selected articles were major anxiety, depression and overall psychiatric disorders.

Altogether 13 articles reported prevalence, six reported service delivery, six on management and another seven on depression as comorbid condition. Only 5 of the 13 prevalence studies published in international journals provided adequate details of methods. Among the articles reported prevalence, 9 studies were community based studies. Diagnosis in all the 5 studies was either made by a psychiatrist or a trained worker using validated instruments, and was considered as good quality. Only 2 studies [11,12] discussed the generalizability of their findings along with the study limitations. We did not find any prospective study presenting the natural course of the disorder or any rigorously controlled study of any intervention.

### Prevalence of mental disorders among adults

Table 1 presents summary of 8 cross-sectional studies that reported the prevalence of mental disorders in adult populations with sample sizes ranging from 327 to 13,080 participants.

**Table 1:** **Summary of the studies reported prevalence of mental disorders in adult population**

**Author’s and publication year**	**Year of data collection**	**Setting**	**Place of the study**	**Age range (years), sexes**	**n (sample size)**	**Outcome measures instruments**	**Prevalence**
Chowdhury, 1975 [13]	1974	Urban	F (OPD, Hospital)	≥ 13 (both)	652	NA	31.4%
Alam, 1978 [14]	1976-1977	Urban	F (GP)	All (both)	1,764	NA	29%
Chowdhury et al., 1981 [15]	1978	Rural	C	All (both)	1,181	NA	6.5%
Ara et al., 2001 [19]	2000	Rural	C	18-65 (women)	415	GHQ-60	16.38%
Islam et al., 2003 [16]	1996-1997	Urban	C	≥18 (both)	1,145	SRQ	28%
Karim et al., 2006 [17]	2003	Rural & Urban	C	≥18 (both)	327	SRQ, SCID NP	12.2%
Hosain et al., 2007 [18]	2000	Rural	C	18-60 (both)	766	GHQ-60	16.5%
NIMH, B and WHO, 2007 [12]	2003-2005	Rural & Urban	C	≥18 (both)	13,080	SRQ, SCID	16.1%

NA = Not available, both = male and female, C = Community based study, F = Facility based study, OPD = Out Patient Department, GP = General Practice, GHQ 60 = General Health Questionnaire 60, SRQ = Self-Reporting Questionnaire, SCID = Structured Clinical Interview for Diagnostic and Statistical Manual of Mental Disorders (DSM-IV), SCID NP = Structured Clinical Interview for diagnosis: non-patient version.

Most commonly used screening and diagnostic tools in these studies were Self Reporting Questionnaire (SRQ), General Health Questionnaire (GHQ), and Structured Clinical Interview for Diagnostic and Statistical Manual of Mental Disorders (SCID). However, 3 articles published in local journals did not clarify type of tools used. The earliest preliminary study conducted in urban setting back in 1975 reported 31% of out-patients had pure psychogenic conditions [13]. Later a general practice (GP) urban setting revealed 29% of the patients were suffering the same [14]. A community based rural study reported 3.6% psychiatric disorders and 2.9% both psychiatric and physical disorders with depression and anxiety being the most common condition [15]. Later an urban community-based study reported psychiatric disorders among 28% of the participants as diagnosed by a psychiatrist [16]. Another study reported an overall prevalence of mental disorders among 12.2% respondents but more females than males were affected (13.9% vs. 10.2%) [17]. A rural community-based study showed an overall prevalence of psychiatric disorders as 16.5%; notably, half of the sufferers had depressive disorders (8%) and a third had anxiety disorders (5%) [18]. On the other hand, another study on females in a rural setting reported 16.4% had mental disorders with depression being the single most common disorder (8.9%) [19]. The first national survey on mental health [12] conducted between 2003 and 2005 documented that 16.1% of the adult population had mental disorders and the prevalence was higher in women than men [12]. Overall, the literature evidence suggest the prevalence of mental disorders ranges from 6.5 to 31.0% among adults depending on the community or clinic setting, and women seemed to be more vulnerable.

### Prevalence of mental disorders among children

Table 2 shows the summary of articles reporting prevalence of mental disorders in children. Sample sizes of the studies varied from 210 to 3,564. Earliest report among urban primary school children revealed 13.4% had some type of behavioral disorder, with boys being twice more affected than girls (20.4 vs. 9.9%) [20]. However, a study among socially disadvantaged (urban slum) children, reported 22.9% had some form of psychiatric disorder with slightly lower prevalence in boys than girls (20.0% in boys and 25.5% in girls) [21]. Mullick & Goodman used Development and Well- Being Assessment (DAWBA) questionnaire, and previously validated Strengths and Difficulties Questionnaire (SDQ) tools [22] in their study and found overall prevalence of 15.2% in different settings (rural, urban and urban slum) with the highest prevalence in the urban slum (19.5%) [23]. Another study found 14.6% children with behavioral problems as reported by the parents in rural Bangladesh [24]. Another more recent community-based study reported prevalence of mental disorder among 18.4% of the children [11].

**Table 2:** **Summary of the studies reported prevalence of mental disorders in children**

**Author’s and publication year**	**Year of data collection**	**Setting**	**Place of the study**	**Age range (years), sexes**	**n (sample size)**	**Outcome measures instruments**	**Prevalence**
Rabbani et al., 1999 [20]	1994	Urban school	S	NA (both)	1,288	Rutter’s B2 Scale	13.4%
Jahan, 2004 [21]	1997-1998	Urban slum	S	10-16 (both)	210	Semi- structured questionnaire	22.9%
Mullick, 2005 [23]	2002-2004	Rural, Urban and Urban Slum	C	5-10 (both)	922	SDQ DAWBA	15.2%
Khan et al., 2008 [24]	2001-2003	Rural	C	2-9 (both)	453	TQ	14.6%
Rabbani et al., 2009 [11]	2009	Rural & Urban	C	5-17 (both)	3,564	RQC	18.4%

NA = Not available, both = male and female, C = Community based study, F = Facility based study, S = School based study, UCEP = Underprivileged Children’s Education Programme, Rutter’s B2 Scale = Rutter’s Behaviour Scale (B2), SDQ = Strengths and Difficulties Questionnaire, DAWBA = Development and Well-Being Assessment, TQ = Ten Questions, RQC = Reporting Questionnaire for Children.

### Depression and comorbidity

Depression is a common comorbid condition with chronic diseases [25]. Table 3 presents the summary of the seven articles reported depression as comorbidity. Five of the studies were hospital-based and the other two were community-based. Six of the studies used cross-sectional and one used case–control study design [26]. An urban hospital-based study reported that among 47% patients with stroke and 54% of cancer patients had depressive episode [27]. Another hospital-based study found 56.6% of cancer patients with major depressive disorders [28]. An urban facility-based study on outpatients reported 16% with purely psychiatric illness and about 3% of the total or 18.2% of purely psychiatric disorders had major depressive disorders [29]. A case control study in rural population concluded that newly diagnosed diabetic patients were four times more likely to have depressive symptoms than those without diabetes and females were more vulnerable than males [26]. Another recent study found depressive symptoms among 34% of diabetes outpatients [30]. One study which was a part of a larger longitudinal epidemiological study on diabetes in rural Bangladesh [31] found 15.3% of the participants with depression [32]. An urban facility-based study reported presence of depression in one in every three diabetes patients [33].

**Table 3:** **Summary of the studies reported depression as co-morbid condition**

**Author’s and publication year**	**Year of data collection**	**Setting**	**Place of the study**	**n (sample size)**	**Outcome measures instruments**	**Disease (n)**	**Comorbidity (n)**
Karim et al., 2001 [27]	1995-98	Urban	F (Hospital)	128	ICD 10 criteria; Multipoint questionnaire	Stroke (32) Cancer (50)	Depression (15) Depression (27)
Chowdhury et al., 2007 [28]	2007	Urban	F (Hospital)	100	DSM-IV criteria; Structured questionnaire	Cancer (100)	Depression (30)
Ali et al., 2007 [29]	2004-2006	Urban	F (Hospital)	415	DSM-IV criteria GHQ-12	Illness (415)	Depression (12)
Asghar et al., 2007 [26]	2004	Rural	C	952	MADRS	Diabetes (184)	Depression (55)
Roy et al., 2012 [30]	2010-2011	Urban and suburban	F (OPD, clinic)	417	WHO-5 and PHQ- 9	Diabetes (417)	Depression (142)
Bhowmik et al., 2012 [32]	2009	Semi urban	C	2293	MADRS	Diabetes (181)	Depression (57)
Rahman et al., 2011 [33]	2009	Urban	F (Hospital)	178	CES-D	Diabetes (178)	Depression (62)

C = Community based study, F = Facility based study, ICD 10 = International Statistical Classification of Diseases and Related Health Problems, DSM- IV = Diagnostic and Statistical Manual of Mental Disorder-4^th^ edition, GHQ 12 **=** General Health Questionnaire, MADRS = Montgomery and Aasberg Depression Rating Scale, WHO-5 = World Health Organization-5 Well Being Index, PHQ- 9 = Patient Health Questionnaire-9, CES-D = Centre for Epidemiological Studies Depression Scale.

### Service delivery and management

In total, twelve articles were found (data not shown) where six [34–39] reported on care seeking pattern, referral and service delivery issues and the other six [40–45] reviewed management of psychiatric disorders in Bangladesh. In a pathway study it was found that only 16% patients came directly to mental health professionals [34]. On the other hand, a significant proportion consulted other care providers including native or religious healers and traditional healers [34,36,43]. Reports indicate that most of the care seekers were referred by old patients, relatives or friends [35,38,43]. Some studies reported long delays in care seeking. A rural study found that two third (65.4%) of the mental patients from rural setting were referred to the hospital 3 months to several years after onset of the disorder [39] whereas in the urban setting the mean delay was about 10 weeks [34]. The main reason for delay was lack of awareness of the seriousness of the condition (69%) [35]. Several articles pointed out the lack of adequate number of psychiatrists given the huge burden of mental health disorders and those available are mostly located in big cities. Notably, mental health services are virtually non-existent at primary care level throughout the country [40,41,44].

## Discussion

We found only a limited number of published studies on the prevalence of psychiatric disorders in Bangladesh. Overall prevalence varied from 6.5 to 31% among adults and from 13.4 to 22.9% among children. Despite wide ranges in prevalence estimates reported, these figures strongly suggest that mental disorders constitute a big public health problem in Bangladesh.

It is worthwhile to mention some of the limitations of the review. Data from the selected articles are not comparable due to differences in settings (clinic- vs. community-based), different assessment tools and the different thresholds used to determine the psychiatric disorders. Therefore the various prevalence estimates available could not be used properly to assess the trend over time. Our review may have been subject to publication and selection bias as we were unable to contact the experts and collect unpublished materials or access any grey literature.

In general, the prevalence estimates of psychiatric disorders are prone to underestimation as majority of patients and their families deny due to strong stigma attached to mental disorders. This limits the number of affected patients seeking health care actively [43].

The prevalence reported by Islam et al. [16] might have underestimated due to focusing on only the major types of psychiatric disorders. There is wide variation between rural communities and also between rural and urban settings [15,18]. The prevalence reported in an urban overcrowded community was much higher as the study included older people who are more prone to mental disorders [16]. This review suggests both rural [15] and urban [13] settings a higher vulnerability of mental disorders among females as compared to males. There is a significantly higher prevalence of mental disorders among economically poor respondents, and specifically among women from large families as reported by Hosain et al. [18]. These findings are consistent with another rural study [19] which reported that social stigma inhibits women from seeking medical treatment for their mental problems. Despite considerable variations in the design of studies, prevalence of psychiatric disorders in adult population is more or less similar to socio-culturally similar settings in neighboring India [46] and Pakistan [47].

The only national survey conducted between 2003 and 2005 illustrated the high burden of mental disorders in Bangladesh [12]. As there is no similar nationally representative mental health survey carried out in recent time, it is not possible to assess the change and to estimate overall need for resources to address the mental health burden. In general, tools for screening and cut-off values used in the reported studies contributed to the variation in the prevalence reported in different articles. However, the problems of underreporting and under-diagnosis of mental disorders are major challenges for the future of psychiatric epidemiology in Bangladesh.

As evident from this review, data on mental disorders among children in Bangladesh are quite scanty. Moreover, the comparison of prevalence studies of childhood psychiatric disorders is challenging due to the heterogeneous nature of samples, screening and diagnostic tools used, and methods of combining information in addition to differences in age distributions which requires standardization for fair comparison. The overall prevalence of psychiatric disorders in a community study by Mullick & Goodman [23] did not differ much with the findings of study by Rabbani et al. [11]. As there are only a handful of child mental health professionals with specialized training in Bangladesh, the vast gap between actual need and available services requires special and immediate attention [23]. Research in socially disadvantaged and underprivileged groups is also needed to improve the diagnosis, treatment and outcomes in those vulnerable groups [21]. Childhood psychiatric disorders were significantly associated with malnutrition [24], rural residence, low education of fathers, and positive family history [11] which all need a multi-sectorial approach to address these neglected areas. The children mental health survey [11] results provided a baseline measure and resources which can be a basis for taking initiatives for further prevalence study as well as creating provision for effective service delivery models.

The interaction between mental disorder and chronic diseases is complex, yet risks and causalities are well-established [48]. Mental disorder may increase the risk for chronic disorders and many chronic disorders can increase an individual’s risk for developing mental disorders, thereby complicating help-seeking, diagnosis, management and prognosis [49].

Current epidemiological evidence suggested at least one third of people with diabetes are suffering from depressive disorders [50,51]. Although in this review we found four articles [26,30,32,33] which reported similarly high prevalence of depression among diabetics. It is reported that more than half of the cancer patients suffer from depression [27,28] which is conceivable given the severity and progressive nature of the disease, high cost and lack of adequate care facilities available. Limited data from South Asian settings reported two- to fivefold increase in the prevalence rates of depression in people with diabetes compared to people without diabetes [52–54]. Depressions intensify symptom presentation and interfere with the physical treatment [27]. Psychiatric morbidity is considerable among the cancer patients that need to be addressed with additional treatment and support [28]. These findings suggest a need for further rigorous study of chronic diseases and mental health for optimizing treatment of both conditions using sound methodologies as well as validated screening and diagnostic tools.

This review confirms inadequate care seeking as well as poor service delivery for mental health disorders. Referrals of patient with mental disorders to mental health specialists by the general practitioners or other health care providers are almost non-existent. The referral is also hampered due to superstitious beliefs related to psychiatric disorders. These are seen as triggered by evil influences while this leads to seeking remedies from traditional healers. These potentially harmful practices can be minimized through mass awareness [34,39] and development and implementation of mental health guidelines. This also can be influenced through raising individual level awareness and social mobilization [55]. Denial mental health problems is common among the general population as they perceive these conditions as untreatable. Additional constraint is imposed by lack adequately trained general practitioners and health workers at primary care level. There are no structured and organized mental health services available at primary and even at secondary health care level. Although women are more often sufferers and also more vulnerable to develop psychiatric disorders, they are more neglected than males in receiving care. This is more likely the result of a male-dominated culture in Bangladesh [38]. Therefore, the access to mental health services need to be made more accessible by the women at all levels of mental health care service delivery [42]. Although Bangladesh has formally a well-structured three tier health care delivery system [56], due to dearth of mental health professionals and poor logistic support, this existing system is not functioning well for mental health conditions [57].

Management of psychotic depression requires treatment from psychiatrists who are mainly available in the tertiary care hospitals in major cities [40]. Primary care services lack adequately trained personnel to identify and treat depression as a single condition or in association with particular chronic disorders. Although training and services to address psychiatric conditions are gradually increasing, vast majority of mental health patients yet to get the benefit of such initiatives as they have limited access [45,57]. Most of the psychotropic medications are available in Bangladesh yet psychotherapy is hardly available. Bangladesh lacks a mental health act although a draft bill is in the final stage but is yet to be approved by the authorities.

## Conclusions

This review showed that researches on mental disorders are not at satisfactory level in Bangladesh given the magnitude of the problem. To improvise the mental health services in Bangladesh, further well-designed epidemiological and clinical research are needed. Public education and awareness campaigns on mental health conditions may be undertaken to ameliorate misconceptions.
